# Reduced expression of ezrin in urothelial bladder cancer signifies more advanced tumours and an impaired survival: validatory study of two independent patient cohorts

**DOI:** 10.1186/1471-2490-14-36

**Published:** 2014-05-12

**Authors:** Gustav Andersson, Christoffer Wennersten, Alexander Gaber, Karolina Boman, Björn Nodin, Mathias Uhlén, Ulrika Segersten, Per-Uno Malmström, Karin Jirström

**Affiliations:** 1 Department of Clinical Sciences, Oncology and Pathology, Lund University, Skåne University Hospital, Lund 221 85, Sweden; 2 Science for Life Laboratory, Royal Institute of Technology, Stockholm 171 21, Sweden; 3 School of Biotechnology, AlbaNova University Center, Royal Institute of Technology, Stockholm 106 91, Sweden; 4 Department of Surgical Sciences, Uppsala University, Uppsala 751 85, Sweden

**Keywords:** Ezrin, Urothelial bladder cancer, Prognosis

## Abstract

**Background:**

Reduced membranous expression of the cytoskeleton-associated protein ezrin has previously been demonstrated to correlate with tumour progression and poor prognosis in patients with T1G3 urothelial cell carcinoma of the bladder treated with non-maintenance Bacillus Calmette-Guérin (n = 92), and the associations with adverse clinicopathological factors have been validated in another, unselected, cohort (n = 104). In the present study, we examined the prognostic significance of ezrin expression in urothelial bladder cancer in a total number of 442 tumours from two independent patient cohorts.

**Methods:**

Immunohistochemical expression of ezrin was evaluated in tissue microarrays with tumours from one retrospective cohort of bladder cancer (n = 110; cohort I) and one population-based cohort (n = 342; cohort II). Classification regression tree analysis was applied for selection of prognostic cutoff. Kaplan-Meier analysis, log rank test and Cox regression proportional hazards’ modeling were used to evaluate the impact of ezrin on 5-year overall survival (OS), disease-specific survival (DSS) and progression-free survival (PFS).

**Results:**

Ezrin expression could be evaluated in tumours from 100 and 342 cases, respectively. In both cohorts, reduced membranous ezrin expression was significantly associated with more advanced T-stage (p < 0.001), high grade tumours (p < 0.001), female sex (p = 0.040 and p = 0.013), and membranous expression of podocalyxin-like protein (p < 0.001 and p = 0.009). Moreover, reduced ezrin expression was associated with a significantly reduced 5-year OS in both cohorts (HR = 3.09 95% CI 1.71-5.58 and HR = 2.15(1.51-3.06), and with DSS in cohort II (HR = 2.77, 95% CI 1.78-4.31). This association also remained significant in adjusted analysis in Cohort I (HR1.99, 95% CI 1.05-3.77) but not in Cohort II. In pTa and pT1 tumours in cohort II, there was no significant association between ezrin expression and time to progression.

**Conclusions:**

The results from this study validate previous findings of reduced membranous ezrin expression in urothelial bladder cancer being associated with unfavourable clinicopathological characteristics and an impaired survival. The utility of ezrin as a prognostic biomarker in transurethral resection specimens merits further investigation.

## Background

In 2008 there were 386 000 estimated new cases of bladder cancer in the world, and approximately 150 000 individuals died from their disease [1]. Bladder cancer is the fourth most common cancer among men in the USA [2].

Standard treatment for non-muscle-invasive carcinoma is transurethral resection of the bladder (TURB), with or without intravesical instillation of bacillus Calmette-Guérin (BCG), to prevent recurrence and progression. In contrast, muscle-invasive carcinoma is treated more aggressively with neoadjuvant chemotherapy and cystectomy [3,4]. However, non-muscle-invasive urothelial carcinoma has a high risk of recurrence and a substantial risk of progression [5], and muscle-invasive carcinoma is associated with a high mortality, despite aggressive treatment [3,6]. Hence, there is a great need for additional biomarkers to predict the risk of recurrence and progression into muscle invasive carcinoma for patients with early stage tumours.

Loss of expression of the membrane-cytoskeletal linking protein ezrin was initially demonstrated to be associated with tumour progression and poor prognosis in patients with T1G3 tumours treated with non-maintenance BCG (n = 92) [7]. In another recent study comprising 104 tumours of different stages and grades, loss of membranous ezrin expression was found to correlate with higher grade and stage, and invasiveness, but the associations with disease progression and survival were not reported [8]. The protein ezrin is closely related to two other membrane associated proteins, radixin and moesin, all three together named ERM proteins. All these proteins are important for regulation of cell adhesion and in the linkage between membrane proteins and the cortical cytoskeleton, thus affecting cell survival, migration and invasion, all factors contributing to tumour progression and development [9-11]. ERM proteins are inactive in the cytoplasm, and activated by binding to the cell membrane [10]. Ezrin is expressed in a variety of cancers [12] and the prognostic value of ezrin expression seems to differ in different cancer forms. In several cancer forms, high expression of ezrin has been associated with more aggressive tumours [13-19], whereas in serous ovarian carcinoma, lost expression of ezrin correlated with a worse prognosis [20], similar to the findings in bladder cancer [7].

The aim of this study was to further evaluate the utility of ezrin as a prognostic biomarker in two independent patient cohorts comprising a total number of 442 cases. Given previous findings of an *in vitro* interaction of ezrin with podocalyxin-like protein (PODXL), an established mediator of metastasis [21], and our recent results demonstrating that membranous PODXL expression is an independent predictor of tumour progression and poor prognosis in urothelial bladder cancer [22], we also examined the correlations between tumour-specific expression of ezrin and PODXL.

## Methods

### Patients

#### Cohort I

This cohort is a consecutive cohort of all patients with a first diagnosis of urothelial bladder cancer in the Department of Pathology, Skåne University Hospital, Malmö, from Oct 1st, 2002 until Dec 31st, 2003, for whom archival TURB specimens could be retrieved (n = 110). The cohort includes 80 (72.7%) men and 30 (27.3%) women with a median age of 72.86 (39.25-89.87) years. Information on vital status was obtained from the Swedish Cause of Death Registry up until Dec 31st 2010. Follow-up started at date of diagnosis and ended at death, emigration or Dec 31st 2010, whichever came first. Median follow-up time was 5.92 years (range 0.03-8.21) for the full cohort and 7.71 years (range 7.04-8.21) for patients alive (n = 48) at Dec 31st 2010. Fortyeight patients (43.6%) died within 5 years.

The distribution of T-stage was 48 (43.6%) pTa, 24 (21.8%) pT1, 37 (33.8) pT2 and 1 (0.9%) pT3. Eighteen (16.4%) tumours were Grade I, 34 (30.9%) Grade II and 58 (52.7%) Grade III. The cohort has also been described previously [22]. Permission for this study was obtained from the Ethics Committee at Lund University.

#### Cohort II

This cohort includes 344 patients from a prospective cohort of patients with newly diagnosed urothelial bladder cancer at Uppsala University Hospital from 1984 up until 2005. TURB specimens have been collected retrospectively and the predominant group of pTa tumours reduced to include 115 cases. Progression-free survival (PFS), overall survival (OS) and disease-specific survival (DSS) were calculated from the date of surgery to date of event or last follow-up. Progression was defined as shift of the tumour into a higher stage. Median time to progression for patients with non-muscle invasive disease was 18.0 months (range 2.0-55.0). Follow-up time for non-recurrent and non-progressing cases were ≥4 and ≥5-years, respectively. The cohort has been described previously [22,23]. Permission for this study was obtained from the Ethics Committee at Uppsala University.

### Tissue microarray construction

All tumours were histopathologically re-evaluated and classified according to the WHO grading system of 2004 by a board certified pathologist. Tissue microarrays (TMAs) were constructed as previously described [22,23] using a semi-automated arraying device (TMArrayer, Pathology Devices, Westminister, MD, USA). All tumour samples were represented in duplicate tissue cores (1 mm).

### Immunohistochemistry and staining evaluation

For immunohistochemical analysis, 4 μm TMA-sections were automatically pre-treated using the PT Link system and then stained in an Autostainer Plus (DAKO; Glostrup, Copenhagen, Denmark) with a polyclonal, monospecific antibody; HPA021616, Atlas Antibodies AB, diluted 1:1500. The specificity of the antibody has been confirmed by immunofluorescence, Western blotting and protein arrays (http://www.proteinatlas.org). Ezrin expression was annotated in accordance with previous studies [7], whereby the percentage of cancer cells with membranous protein sub-localization and the intensity of cytoplasmic staining, ranging from 0–3 (negative, weak, moderate, strong), was denoted. When evaluating cytoplasmic staining the dominating intensity for each core was determined. A mean value of the two samples from each tumour was used in the statistical analyses. The staining was evaluated by three independent observers, including one board certified pathologist, who were blinded to clinical and outcome data and every sample was re-evaluated once. Omission of primary antibody was used as a negative control, normal colonic mucosa as positive external control and lymphocytes as an internal positive control. Discrepant cases were re-evaluated once again and discussed in order to reach consensus.

Immunohistochemical expression of PODXL had been performed as previously described, whereby the presence of membranous expression was demonstrated to be prognostic [22].

### Statistics

Non-parametric Mann–Whitney U or Kruskal-Wallis tests were applied for analysis of the correlations between membranous ezrin expression and clinicopathological characteristics. Classification and regression tree (CRT) analysis [24] was used to assess optimal prognostic cut-offs for ezrin expression. Receiver operating characteristics (ROC) curve analysis was also applied to verify the CRT-derived cutoffs. Kaplan-Meier analysis and log rank test were used to illustrate differences in 5-year overall survival (OS) and disease-specific survival (DSS) in strata according to high and low ezrin expression. Cox regression proportional hazards modeling was used to estimate the impact of ezrin expression on 5-year OS in both univariable and multivariable analysis, adjusted for age, sex, T-stage and grade. All tests were two sided. P-values <0.05 was considered significant. All statistical analyses were performed using IBM SPSS Statistics version 20.0 (SPSS Inc., Chicago, IL, USA).

## Results

### Distribution of ezrin expression and its association with clinicopathological characteristics

**Figure 1:**
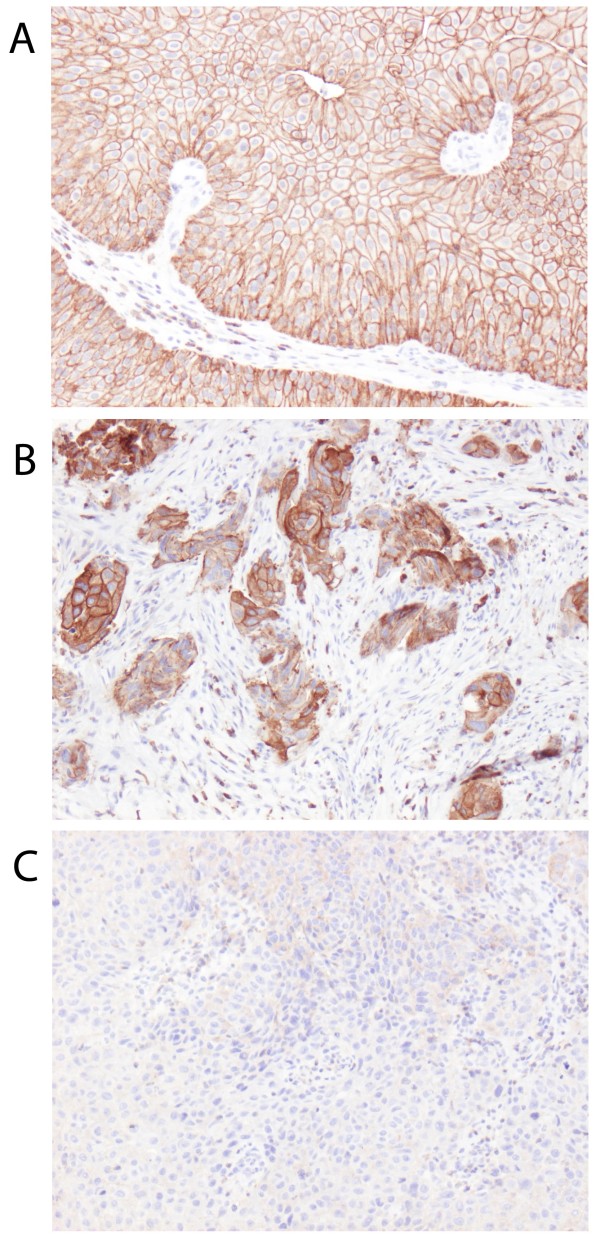
**Sample immunohistochemical images of ezrin expression.** Images (20x magnification) representing tumours with **(A)** nearly 100%, **(B)** approximately 50% and **(C)** negative ezrin expression.

**Figure 2:**
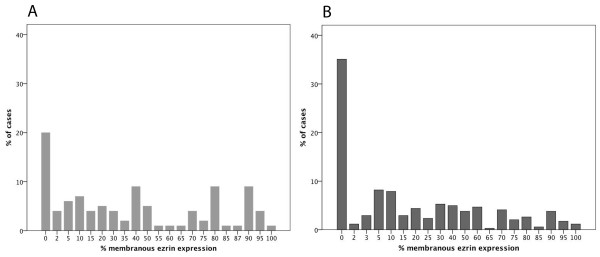
**Distribution of ezrin expression in two independent patient cohorts.** Bar charts visualizing the distribution of different percentages of ezrin expression in **(A)** cohort I (n = 100) and **(B)** cohort II (n = 342).

Following antibody optimisation and staining, ezrin expression could be evaluated in tumours from 100/110 (90.9%) cases in Cohort I and 342/344 (99.4%) cases in Cohort II. Lost cases were either a result of complete tissue loss during IHC preparation or an insufficient quantity of tumour tissue. Sample IHC images are shown in Figure 1 and the distribution of membranous ezrin expression in both cohorts is shown in Figure 2. There was no obvious heterogeneity regarding membranous or cytoplasmic ezrin expression between duplicate cores.

As shown in Table 1, analyses of the relationship between membranous staining and established clinicopathological factors revealed strong, significant associations between reduced membranous ezrin expression and more advanced T-stage and high grade tumours in both cohorts (p < 0.001 for all). Moreover, there was a significant association between female gender and reduced membranous ezrin expression in both cohorts (p = 0.040 and p = 0.013). No associations were found between membranous ezrin expression and age. Cytoplasmic ezrin expression was not associated with any clinicopathological factors or with membranous ezrin expression (data not shown). In light of the significant associations of ezrin expression with female sex, the distribution of grade and T-stage according to sex was aslo analyzed, whereby it was found that grade did not differ by sex in neither cohort and that the distribution of T-stages was equal in both sexes in cohort I, but not in cohort II, where stage II-IV tumours were more common in women (p = 0.030).

Reduced ezrin expression was also significantly associated with the presence of membranous PODXL expression in both cohorts (p < 0.001 and p = 0.009).

**Table 1:** Associations of membranous ezrin expression with clinicopathological and investigative parameters in two independent patient cohorts of urothelial bladder cancer

		**Cohort I (n = 100)**			**Cohort II (n = 342)**	
**Factor**	**n (%)**	**Ezrin expression**		**n (%)**	**Ezrin expression**	
		**median (range)**	***p-value***		**median (range)**	***p-value***
**Age**						
≤ average	46 (46.9)	30.9 (0.0-100)	0.222	157 (45.9)	10.0 (0.0-100)	0.136
>average	54 (54.0)	35.0 (0.0-95.0)		185 (54.1)	10.0 (0.0-100)	
**Gender**						
Female	25 (25.9)	10.0 (0.0-90.0)	0.040	82 (24.0)	5.00 (0.0-90.0)	0.013
Male	75 (75.0)	40.0 (0.0-100)		260 (76.0)	10.0 (0.0-100)	
**T-stage**						
Ta	41 (41.0)	75.0 (0.0-100)	<0.001	115 (33.6)	30.0 (0.0-100)	<0.001
T1	22 (22.0)	25.0 (0.0-95.0)		116 (33.9)	10.0 (0.0-95.0)	
T2-3	37 (37.0)	2.0 (0.0-80.0)		111 (32.5)	0.0 (0.0-60.0)	
**Grade**						
Low	44 (44.0)	72.5 (0.0-100)	<0.001	82 (24.0)	40.0 (0.0-100)	<0.001
High	56 (56.0)	10.0 (0.0-90.0)		260 (76.0)	5.0 (0.0-95.0)	
**PODXL expression**						
Negative	78 (78.8)	40.0 (0.0-100)	<0.001	306 (89.7)	10.0 (0.0-100)	0.009
Positive	21 (21.0)	5.00 (0–70.0)		35 (10.2)	(0.0-0.80)	

### Impact of membranous ezrin expression on survival

CRT analysis determined optimal prognostic cut-offs at 17.5% and 27.5% positive ezrin expression in cohort I and II, respectively, for 5-year OS in cohort I and II (Additional file 1A and 1B). In cohort II, an optimal cutoff for DSS was set at 12.5% (Additional file 1C). The same optimal prognostic cutoffs were obtained by ROC analysis (data not shown).

**Figure 3:**
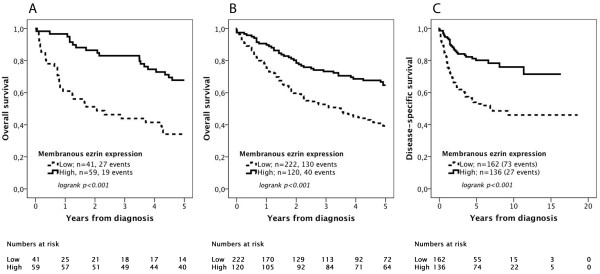
**Kaplan-Meier estimates of bladder cancer specific survival and 5-year overall survival.** Kaplan-Meier analysis of 5-year overall survival in **(A)** cohort I and **(B)** cohort II, and **(C)** disease-specific survival in cohort II.

As demonstrated in Figure 3, loss of ezrin expression was significantly associated with a reduced 5-year OS in cohort I (logrank p < 0.001, Figure 3A) and in cohort II (logrank p < 0.001, Figure 3B). In cohort II, low ezrin expression was also significantly associated with an impaired DSS (logrank p < 0.001, Figure 3C).

The associations between ezrin expression and survival were confirmed in unadjusted Cox regression analysis (Table 2). In adjusted analysis, reduced ezrin expression remained an independent predictor of a significantly reduced 5-year OS in Cohort I (HR = 1.99 (95% CI = 1.05-3.77). In cohort II, however, loss of ezrin expression did not remain prognostic after adjustment for established prognostic factors, neither for 5-year OS nor for DSS (Table 2).

**Table 2:** Relative risks of death from disease and overall death within 5 years according to clinicopathological factors and ezrin expression in two independent patient cohorts

	**Cohort I**			**Cohort II**					
	**Risk of death within 5 years**				**Risk of death from disease**			**Risk of death within 5 years**	
		**Unadjusted**	**Adjusted**		**Unadjusted**	**Adjusted**		**Unadjusted**	**Adjusted**
	**n (events)**	**HR (95% CI)**	**HR (95% CI)**	**n (events)**	**HR (95% CI)**	**HR (95% CI)**	**n (events)**	**HR (95% CI)**	**HR (95% CI)**
**Age**									
Continuous	100(46)	1.05(1.02-1.08)	1.05(1.02-1.08)	300(100)	1.05(1.03-1.07)	1.05(1.02-1.07)	342(170)	1.06(1.05-1.08)	1.07(1.05-1.08)
**Gender**									
Female	25(11)	1.00	1.00	71(28)	1.00	1.00	82(40)	1.00	1.00
Male	75(35)	0.96(0.49-1.89)	1.12(0.54-2.31)	227(72)	0.79(0.51-1.22)	1.03(0.66-1.61)	260(130)	0.97(0.68-1.38)	1.20(0.83-1.72)
**Stage**									
Ta	41(7)	1.00	1.00	104(13)	1.00	1.00	115(35)	1,00	1.00
T1	22(15)	5.06(2.06-12.46)	2.20(0.73-6.68)	97(25)	2.20(1.13-4.31)	2.17(1.11-4.25)	116(53)	1.63(1.06-2.50)	1.57(1.02-2.40)
T2-4	37(24)	6.15(2.64-14.31)	2.44(0.73-8.19)	97(62)	8.86(4.86-16.16)	8.70(4.76-15.89)	111(82)	4.34(2.91-6.46)	4.36(2.92-6.52)
**Grade**									
Low	44(9)	1.00	1.00	75(7)	1.00	1.00	82(20)	1.00	1.00
High	56(37)	4.71(2.27-9.79)	2.94(1.32-6.53)	223(93)	5.75(2.66-12.39)	1.67(0.64-4.35)	260(150)	3.09(1.94-4.93)	1.20(0.67-2.13)
Ezrin expression*									
High	59(19)	1.00	1.00	136(27)	1.00	1.00	120(40)	1.00	1.00
Low	41(27)	3.09(1.71-5.58)	1.99(1.05-3.77)	162(73)	2.77(1.78-4.31)	1.23(0.75-2.02)	222(130)	2.15(1.51-3.06)	1.24(0.84-1.84)

*High and low expression determined by CRT-analysis; cutoff 17.5% for 5-year OS in Cohort I, 27.5% for for 5-year OS in Cohort II and 12.5% for DSS in cohort II.

Given the strong association between reduced ezrin expression and the presence of membranous PODXL expression, previously demonstrated to be an independent factor of tumour progression and an impaired survival in the herein investigated cohorts [22], we also compared the prognostic ability of ezrin and PODXL in the multivariable model. This revealed that inclusion of PODXL did not alter the prognostic value of ezrin expression, that was retained in Cohort I but not in Cohort II, neither for 5-year OS nor DSS. Of note, PODXL remained an independent prognostic factor in Cohort II, for both 5-year OS and DSS, but not in cohort I (data not shown).

Since the only previous study on the prognostic value if ezrin expression in bladder cancer was performed on tumours from patients with T1 tumours [7], the prognostic value of ezrin expression in subgroups according to T-stage was also examined. However, ezrin expression was not found to be prognostic in any particular T-stage in neither cohort (data not shown). Furthermore, in contrast to PODXL, reduced ezrin expression was not significantly associated with time to progression in non-muscle invasive (pTa and pT1 or pT1) tumours in cohort II (n = 134 or n = 66, data not shown). Of note, the number of cases that had received BCG treatment was only 17 and 7, respectively, in these two patient categories, hence not allowing for analyses of a potential treatment predictive effect of ezrin. There was no significant association between reduced ezrin expression and a more frequent rate of recurrence (data not shown).

There were no significant associations between cytoplasmic ezrin expression and survival in neither of the analysed cohorts, and a combined score of cytoplasmic and membranous ezrin expression did not add prognostic value (data not shown). Since the percentage of membranous staining was similar between duplicate cores, use of best or worst score did, as expected, not improve the prognostic value of ezrin expression (data not shown).

## Discussion

The results from this study demonstrate that loss of membranous ezrin expression in urothelial bladder cancer is strongly associated with a more aggressive tumour phenotype; i.e. higher grade and more advanced tumour stage, and an impaired survival. To our best knowledge, the expression of ezrin in urothelial bladder cancer has only been described in two previous studies; one selected cohort of T1G3 tumours (n = 98) treated with non-maintenance BCG [7], and another unselected cohort (n = 104). In the former study, while correlations of ezrin expression with stage and grade could not be performed, the authors found no associations between ezrin expression and age, sex, substaging, tumour size, focality or the presence of CIS, but that reduced ezrin expression was an independent predictor of progression into muscle-invasive disease and shorter disease- specific survival [7]. In the latter study, loss of ezrin was found to correlate with higher grade and T-stage, and with muscularis propria invasion, but hazard ratios for risk of progression or association with survival were not presented [8].

Thus, the results from our study further validate previous findings of strong significant associations between loss of membranous ezrin expression and more advanced T-stage and high tumour grade in urothelial bladder cancer. Moreover, this study is the first to report the prognostic value of ezrin expression in tumours representing all stages and grades, whereby reduced ezrin expression was found to be associated with a significantly reduced 5-year OS in both examined cohorts, and with a significantly reduced DSS in cohort II. Of note, ezrin expression only retained an independent prognostic value for 5-year OS in the smaller cohort, and neither for 5-year OS nor for DSS in the larger, clinically more well-characterized, cohort. Therefore, further validation of the prognostic value of ezrin expression in additional patient cohorts is warranted. Nevertheless, since tumour stage may be difficult to determine in TURB-specimens, the strong link between loss of ezrin expression and advanced tumour stage found here indicates that assessment of ezrin may be an important surrogate marker for bladder cancer patients at risk of having progressive disease.

In contrast to the findings by Palou et al. [7], we were not able to demonstrate an association between reduced ezrin expression and risk of progression into muscle-invasive disease in pTa-pT1 or pT1-tumours (information only available in cohort II). It should however be pointed out that the number of patients having received BCG-treatment in our study was too small, and since all patients in the study by Palou had received non-maintenance BCG-treatment [7], a potential treatment predictive effect of ezrin cannot be ruled out, and should be taken into consideration in future studies.

The observed significant association between reduced ezrin expression and female sex in both cohorts is noteworthy, not least since the distribution of tumour grades did not differ between sexes in any of the cohorts, and a significant association between female sex and more advanced T-stage could only be found in cohort II. While the risk of bladder cancer is considerably higher in men, there is data indicating an impaired survival from bladder cancer in women [25]. These findings indicate that ezrin may be a relevant investigative biomarker in studies related to the molecular pathological epidemiology of urothelial bladder cancer, in particular studies addressing the influence of sex hormones and reproductive factors on cancer risk and survival.

Despite use of different antibodies for detection of ezrin expression in the two previous studies [7,8] and the present, the results were concordant, which further supports the utility of ezrin as a prognostic and, potentially treatment predictive, biomarker in urothelial bladder cancer. Immunohistochemistry has several advantages compared with other assays, e.g. gene expression analyses, since it is comparatively cheap, can readily be adopted into clinical protocols, and, most importantly, allows for assessment of biomarkers in relation to their subcellular location. In our study, no prognostic value or correlation with clinicopathological factors could be demonstrated for cytoplasmic ezrin expression, which is also in line with previous findings [7,8]. The lack of prognostic value for cytoplasmic ezrin expression is also in agreement with the observation of ERM proteins only being active when bound to the cell membrane and not when located in the cytoplasm [10].

The herein observed inverse association between membranous expression of ezrin and PODXL does not provide evidence of, but may well indicate, a functional link between these proteins in urothelial bladder cancer. This hypothesis is further supported by the previously demonstrated ability of PODXL to form complex with ezrin in breast and prostate cancer cells *in vitro*, thereby inducing phosphorylation of ezrin and changes in its subcellular location, in turn leading to an increased migration and invasion [21]. In light of the apparently contrasting prognostic value and intercorrelation of ezrin and PODXL expression in urothelial bladder cancer, it will be of interest to investigate the existence of a negative functional cooperativity between these proteins in this cancer form.

Of note, although cutoffs selected by CRT-analysis in our study varied somewhat between the cohorts and according to the endpoint, ranging from 12.5% to 27.5% membranous positivity, they still landed closely to the prognostic cutoff determined as the median percentage of ezrin expression at 20% in the study by Palou et al. [7], although all tumours in their study were pT1G3. A different approach was used in the study by Athanasopoulou et al., where four categories of a combined score of percentage and intensity of membranous ezrin immunoreactivity was applied in the statistical analyses [8]. Moreover, in that study, nearly all (103/104) tumours were reported to have positive membranous ezrin expression [8]. Future studies, preferably in the prospective setting, are warranted to determine optimal cutoffs for the potential use of ezrin as a biomarker in clinical practice.

## Conclusions

In summary, the results from this study demonstrate that reduced membranous ezrin expression in urothelial bladder cancer is associated with more advanced tumours and a reduced survival. These findings suggest that ezrin may be a useful prognostic biomarker and possibly aid in tailoring the treatment of patients with non-muscle-invasive carcinoma of the bladder. Further studies are warranted in order to confirm the utility of ezrin as a prognostic biomarker in clinical practice.

## Abbreviations

TMA: Tissue microarray; CRT: Classification regression tree; DSS: Disease-specific survival; OS: Overall survival; PFS: Progression-free survival; HR: Hazard ratio; CI: Confidence interval.

## Competing interests

The authors declare that they have no competing interests.

## Authors’ contributions

GA and CW evaluated the immunohistochemical stainings, performed the statistical analyses and drafted the manuscript. US, PUM and KB collected clinical data. US and BN constructed the TMAs and BN performed the immunohistochemcal stainings. AG assisted with the statistical analysis and helped draft the manuscript. MU contributed with antibody validation. KJ conceived of the study, evaluated the immunohistochemistry, and helped draft the manuscript. All authors read and approved the final manuscript.

## Pre-publication history

The pre-publication history for this paper can be accessed here:

http://www.biomedcentral.com/1471-2490/14/36/prepub

## Supplementary Material

Additional file 1 **Classification regression tree analysis for selection of prognostic cutoffs.** (A) Overall survival in Cohort I, (B) overall survival in cohort II and (C) disease-specific survival in cohort II. Click here for file

## References

[B1] Ferlay J Shin HR Bray F Forman D Mathers C Parkin DM Estimates of worldwide burden of cancer in 2008: GLOBOCAN 2008 Int J Cancer 2010 127 12 2893 2917 10.1002/ijc.25516 21351269

[B2] Jemal A Siegel R Xu J Ward E Cancer statistics, 2010 CA Cancer J Clin 2010 60 5 277 300 10.3322/caac.20073 20610543

[B3] Raghavan D Shipley WU Garnick MB Russell PJ Richie JP Biology and management of bladder cancer N Engl J Med 1990 322 16 1129 1138 10.1056/NEJM199004193221607 2181313

[B4] Schenkman E Lamm DL Superficial bladder cancer therapy ScientificWorldJournal 2004 4 1 387 399 15349563 10.1100/tsw.2004.81 PMC5956304

[B5] van der Heijden AG Witjes JA Recurrence, Progression, and Follow-Up in Non–Muscle-Invasive Bladder Cancer Eur Urol Suppl 2009 8 7 556 562 10.1016/j.eursup.2009.06.010

[B6] Knowles MA Molecular subtypes of bladder cancer: Jekyll and Hyde or chalk and cheese? Carcinogenesis 2006 27 3 361 373 10.1093/carcin/bgi310 16352616

[B7] Palou J Algaba F Vera I Rodriguez O Villavicencio H Sanchez-Carbayo M Protein expression patterns of ezrin are predictors of progression in T1G3 bladder tumours treated with nonmaintenance bacillus Calmette-Guerin Eur Urol 2009 56 5 829 836 10.1016/j.eururo.2008.09.062 18926620

[B8] Athanasopoulou A Aroukatos P Nakas D Repanti M Papadaki H Bravou V Decreased ezrin and paxillin expression in human urothelial bladder tumors correlate with tumor progression Urol Oncol 2013 31 6 836 842 10.1016/j.urolonc.2011.07.003 21868260

[B9] Mangeat P Roy C Martin M ERM proteins in cell adhesion and membrane dynamics Trends Cell Biol 1999 9 5 187 192 10.1016/S0962-8924(99)01544-5 10322453

[B10] Bretscher A Edwards K Fehon RG ERM proteins and merlin: integrators at the cell cortex Nat Rev Mol Cell Biol 2002 3 8 586 599 10.1038/nrm882 12154370

[B11] Curto M McClatchey AI Ezrin…a metastatic detERMinant? Cancer Cell 2004 5 2 113 114 10.1016/S1535-6108(04)00031-5 14998486

[B12] Bruce B Khanna G Ren L Landberg G Jirstrom K Powell C Borczuk A Keller ET Wojno KJ Meltzer P Baird K McClatchey A Bretscher A Hewitt SM Khanna C Expression of the cytoskeleton linker protein ezrin in human cancers Clin Exp Metastasis 2007 24 2 69 78 10.1007/s10585-006-9050-x 17370041

[B13] Li Q Gao H Xu H Wang X Pan Y Hao F Qiu X Stoecker M Wang E Wang E Expression of ezrin correlates with malignant phenotype of lung cancer, and in vitro knockdown of ezrin reverses the aggressive biological behavior of lung cancer cells Tumour Biol 2012 33 5 1493 1504 10.1007/s13277-012-0400-9 22528947

[B14] Tan J Zhang C Qian J Expression and significance of Six1 and Ezrin in cervical cancer tissue Tumour Biol 2011 32 6 1241 1247 10.1007/s13277-011-0228-8 21874375

[B15] Ohtani K Sakamoto H Rutherford T Chen Z Satoh K Naftolin F Ezrin, a membrane-cytoskeletal linking protein, is involved in the process of invasion of endometrial cancer cells Cancer Lett 1999 147 1–2 31 38 10660086 10.1016/s0304-3835(99)00272-4

[B16] Elliott BE Meens JA SenGupta SK Louvard D Arpin M The membrane cytoskeletal crosslinker ezrin is required for metastasis of breast carcinoma cells Breast Cancer Res 2005 7 3 R365 R373 10.1186/bcr1006 15987432 PMC1143558

[B17] Weng WH Ahlen J Astrom K Lui WO Larsson C Prognostic impact of immunohistochemical expression of ezrin in highly malignant soft tissue sarcomas Clin Cancer Res 2005 11 17 6198 6204 10.1158/1078-0432.CCR-05-0548 16144921

[B18] Jin J Jin T Quan M Piao Y Lin Z Ezrin overexpression predicts the poor prognosis of gastric adenocarcinoma Diagn Pathol 2012 7 135 10.1186/1746-1596-7-135 23039327 PMC3502371

[B19] Kang YK Hong SW Lee H Kim WH Prognostic implications of ezrin expression in human hepatocellular carcinoma Mol Carcinog 2010 49 9 798 804 20572160 10.1002/mc.20653

[B20] Moilanen J Lassus H Leminen A Vaheri A Butzow R Carpen O Ezrin immunoreactivity in relation to survival in serous ovarian carcinoma patients Gynecol Oncol 2003 90 2 273 281 10.1016/S0090-8258(03)00262-2 12893187

[B21] Sizemore S Cicek M Sizemore N Ng KP Casey G Podocalyxin increases the aggressive phenotype of breast and prostate cancer cells in vitro through its interaction with ezrin Cancer Res 2007 67 13 6183 6191 10.1158/0008-5472.CAN-06-3575 17616675

[B22] Boman K Larsson AH Segersten U Kuteeva E Johannesson H Nodin B Eberhard J Uhlen M Malmstrom PU Jirstrom K Membranous expression of podocalyxin-like protein is an independent factor of poor prognosis in urothelial bladder cancer Br J Cancer 2013 108 11 2321 2328 10.1038/bjc.2013.215 23652315 PMC3681027

[B23] Boman K Segersten U Ahlgren G Eberhard J Uhlen M Jirstrom K Malmstrom PU Decreased expression of RNA-binding motif protein 3 correlates with tumour progression and poor prognosis in urothelial bladder cancer BMC Urol 2013 13 1 17 10.1186/1471-2490-13-17 23565664 PMC3635919

[B24] Breiman L Classification and regression trees 1993 New York, N.Y.: Chapman & Hall

[B25] Jung KW Park S Shin A Oh CM Kong HJ Jun JK Won YJ Do female cancer patients display better survival rates compared with males? Analysis of the Korean national registry data, 2005–2009 PLoS One 2012 7 12 e52457 10.1371/journal.pone.0052457 23300677 PMC3530449

