# Supplementary figures and images for: Reduced expression of ezrin in urothelial bladder cancer signifies more advanced tumours and an impaired survival: validatory study of two independent patient cohorts
Source: BMC Urol. 2014 May 12;14:36. doi: 10.1186/1471-2490-14-36 (PMC4049499; doi:10.1186/1471-2490-14-36)

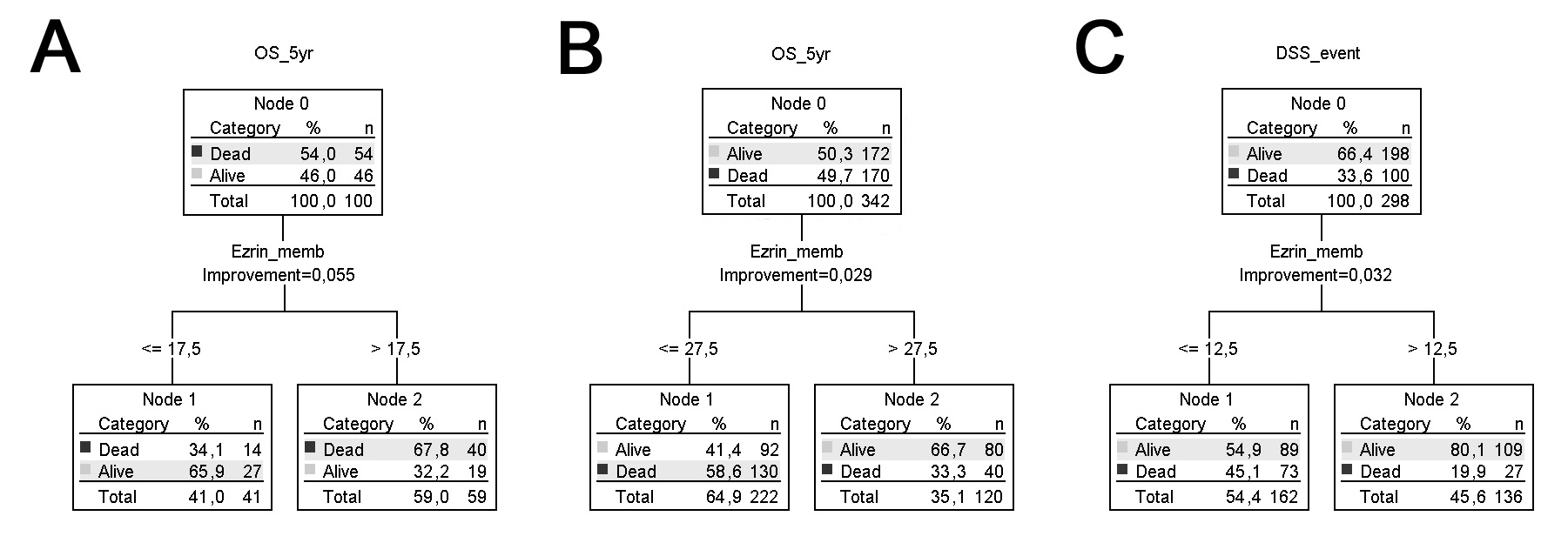

Supplement: Additional file 1 — Classification regression tree analysis for selection of prognostic cutoffs. (A) Overall survival in Cohort I, (B) overall survival in cohort II and (C) disease-specific survival in cohort II. [file 1471-2490-14-36-S1.jpeg]
